# Time-resolved 3D imaging opportunities with XMPI at ForMAX

**DOI:** 10.1107/S1600577525011038

**Published:** 2026-01-21

**Authors:** Julia Katharina Rogalinski, Zisheng Yao, Yuhe Zhang, Zhe Hu, Korneliya Gordeyeva, Tomas Rosén, Daniel Söderberg, Andrea Mazzolari, Jackson da Silva, Vahid Haghighat, Samuel A. McDonald, Kim Nygård, Eleni Myrto Asimakopoulou, Pablo Villanueva-Perez

**Affiliations:** a Synchrotron Radiation Research and NanoLund Lund University Lund Sweden; b Department of Fibre and Polymer Technology Royal Institute of Technology Stockholm Sweden; c Wallenberg Wood Science Center Royal Institute of Technology Stockholm Sweden; d INFN Section of Ferrara, Ferrara, Italy; e MAX IV Laboratory Lund University Lund Sweden; Paul Scherrer Institut, Switzerland

**Keywords:** time-resolved 3D imaging, X-ray imaging, X-ray multi-projection imaging, MAX IV, ForMAX beamline

## Abstract

X-rays are commonly used in imaging experiments due to their penetration power, which enables non-destructive resolution of internal structures in samples that are opaque to visible light. Time-resolved X-ray tomography is the state-of-the-art method for obtaining volumetric 4D (3D + time) information by rotating the sample and acquiring projections from different angular viewpoints over time. This method enables studies to address a plethora of research questions across various scientific disciplines. However, it has several limitations, such as incompatibility with single-shot experiments, challenges in rotating complex sample environments that restrict the achievable rotation speed or range, and the introduction of centrifugal forces that can affect the sample’s dynamics. These limitations can hinder and even preclude the study of certain dynamics. Here, we present an implementation of an alternative approach, X-ray multi-projection imaging (XMPI), which eliminates the need for sample rotation. Instead, the direct incident X-ray beam is split into beamlets using beam splitting X-ray optics. These beamlets intersect at the sample position from different angular viewpoints, allowing multiple projections to be acquired simultaneously. We commissioned this setup at the ForMAX beamline at MAX IV, the first operational diffraction-limited storage ring. We present projections acquired from two different sample systems – fibers under mechanical load and particle suspension in multiphase flow – with distinct spatial and temporal resolution requirements. We demonstrate the capabilities of the ForMAX XMPI setup using the detector’s full analog-to-digital converter range for the relevant sample-driven spatiotemporal resolutions: (i) at least 12.5 kHz frame rates with 4 µm pixel sizes (fibers) and (ii) 40 Hz acquisitions with 1.3 µm pixel sizes (multiphase flows). The presented setup and results form the basis for a permanent XMPI endstation at ForMAX, offering flexibility to adapt to the spatiotemporal requirements of the studied dynamics.

## Abstract 2

The first implementation of X-ray multi-projection imaging (XMPI) at the ForMAX beamline (MAX IV) enables the acquisition of multiple projections simultaneously without requiring sample rotation. This approach facilitates volumetric studies of fast dynamics at frame rates of 12.5 kHz with micrometre resolution, with the potential of even higher speeds, surpassing the limitations of state-of-the-art methods like time-resolved tomography.

## 1. Introduction

Advancements in accelerator technologies have prompted the development of the diffraction-limited storage ring (DLSR), also known as fourth-generation synchrotron light sources. Using multibend achromats, the emittance of the electron beam can be significantly decreased, leading to an increase in brilliance by up to several orders of magnitude compared with their predecessors (Eriksson *et al.*, 2014 ▸; Raimondi & Liuzzo, 2023 ▸). As a consequence, the flux density, *i.e.* the number of photons per unit of area and time, is increased, thereby creating new opportunities to achieve higher spatiotemporal resolution in X-ray imaging experiments (Villanova *et al.*, 2017 ▸; García-Moreno *et al.*, 2023 ▸; Yao *et al.*, 2024 ▸).

The MAX IV Laboratory in Lund, Sweden, is the first operational DLSR worldwide, providing beamlines with unprecedented flux capabilities. The ForMAX beamline, designed for scattering and imaging experiments, delivers synchrotron radiation through an undulator. A full undulator harmonic yields high spectral flux density, *i.e.* number of photons per unit of time per unit of area per bandwidth, within a well defined energy bandwidth, in contrast to broader spectra generated, for example, by wigglers. The narrow energy bandwidth enables quantitative analysis of material density and composition, avoiding complications introduced by polychromatic beams (Maire & Withers, 2014 ▸). ForMAX delivers an exceptionally high photon flux of approximately 5 × 10^14^ photons s^−1^ (Nygård *et al.*, 2024 ▸), and consequently high photon flux density. This capability supports highly time-resolved imaging experiments, including time-resolved tomography, the state-of-the-art method for acquiring time-resolved volumetric information in a non-destructive manner (Yao *et al.*, 2024 ▸).

Synchrotron X-ray tomography is performed by rotating a sample relative to an incident beam, thereby acquiring projections (tomograms) of the sample. A detector positioned downstream of the sample collects a sequence of tomograms, typically over a 180° rotation, which is used to reconstruct the full sample volume. Time-resolved tomography requires the acquisition of a complete tomographic dataset within the temporal resolution of interest. For example, to resolve 10 Hz dynamics, a sample rotation with a speed of at least 10 revolutions per second (10 Hz) is required, assuming one complete 3D dataset every 180° and Nyquist sampling. The higher the temporal resolution, the more stringent the requirements become for rotation stages, detector efficiency, photon flux, and the design for *in situ* and operando sample environments. While technological advancements have improved detectors and X-ray sources, achieving stable high-speed sample rotation remains a significant challenge. Time-resolved tomography has been demonstrated at temporal resolutions up to 1 kHz with a spatial resolution of 8.2 µm, using a highly customized experimental setup to study the evolution of metallic foams (García-Moreno *et al.*, 2021 ▸).

Such advancements, while impactful, underscore the inherent limitations of this technique for time-resolved volumetric imaging of multiple sample categories, due to the fundamental requirement for sample rotation. Rotation-sensitive samples, such as dense liquids and soft materials, have intrinsic dynamics that can be affected by the centrifugal forces generated during rotation, thereby compromising the observations. Moreover, samples requiring complex experimental environments face significant challenges with respect to mechanical stability, cable management and uniform sample visibility, which often affect the quality of the collected data. Samples with above 20 Hz dynamics often require custom rotation stages, which are not widely accessible, cannot serve as a universal solution for all samples and may struggle to exceed 1000 revolutions per second (García-Moreno *et al.*, 2023 ▸). Additionally, single-shot phenomena, such as shock wave propagation (Schropp *et al.*, 2015 ▸) or investigations of defect effects (Kumar *et al.*, 2016 ▸), cannot be captured with conventional tomography on a single sample. These studies rely instead on the possibility of preparing identical sample copies and reproducible processes, which is not always possible. As a result, time-resolved tomography leaves a vast spatiotemporal domain unexplored.

These limitations hinder the progress of numerous scientific and industrial studies. Some indicative examples are studies of (i) multiphase opaque flows through complex geometries (*e.g.* blood flow in veins), where radial forces interfere with natural flow behavior, (ii) mechanical properties of novel, sustainable materials (*e.g.* cellulose foams and wood fibers), which are too delicate and prone to damage under rotation, and (iii) damage mechanisms on materials such as fiber-reinforced polymer composites, which are widely used in engineering applications, where failure modes occur within milliseconds, or less, and require temporal resolutions exceeding 1 kHz.

Alternative methods for acquiring time-resolved volumetric information are required to meet the growing needs of the scientific community. The approach that is followed here is X-ray multi-projection imaging (XMPI) (Hoshino *et al.*, 2013 ▸; Villanueva-Perez *et al.*, 2018 ▸; Duarte *et al.*, 2019 ▸; Bellucci *et al.*, 2023 ▸; Voegeli *et al.*, 2023 ▸; Asimakopoulou *et al.*, 2024 ▸; Voegeli *et al.*, 2024 ▸; Yashiro *et al.*, 2024 ▸; Sumiishi *et al.*, 2024 ▸), a technique that provides multiple, angularly spaced illumination points on the sample position by employing beam splitting optics, enabling volumetric data acquisition without the need for sample rotation. By eliminating the requirement for rotation, XMPI opens new possibilities for studying complex sample environments and sensitive dynamic processes. The employment of XMPI at high spectral flux density facilities such as ForMAX (MAX IV) enables access to previously inaccessible temporal resolutions in systems that are incompatible with tomography, thus allowing the exploration of new physical phenomena.

XMPI has become feasible through advances in fast detector readout schemes, improvements in crystal manufacturing for beam splitting optics, and the development of novel reconstruction algorithms. There remains significant potential to further optimize XMPI by exploring alternative crystal materials with enhanced diffraction efficiency, testing different beam splitting schemes to explore various geometries, and implementing improved mounting and cooling strategies to reduce artifacts caused by heat load (Bellucci *et al.*, 2024 ▸). In parallel, ongoing developments on reconstruction algorithms aim to improve reconstruction quality when only a sparse number of projections is available (Zhang *et al.*, 2025 ▸; Hu *et al.*, 2025 ▸).

The paper introduces an XMPI setup tailored to the ForMAX beamline at MAX IV and demonstrates its use in two scientific applications enabled by XMPI: (i) the loading behavior of wood fibers at different stages of kraft cooking and (ii) particle suspension in multiphase flows. The structure of the paper is as follows. First, we introduce in Section 2 the ForMAX beamline, the XMPI instrumentation and the data processing. In Section 3, we discuss the studied sample processes, the need to address them with XMPI and details of the experimental apparatus. In Section 4, we discuss our results. Finally, in Section 5, we conclude our results, provide an outlook for future improvements and highlight additional scientific applications that could benefit from XMPI at ForMAX.

## 2. Instrumentation

### 2.1. The ForMAX beamline

The ForMAX beamline of MAX IV (Nygård *et al.*, 2024 ▸) was developed to meet the need for structural characterization of hierarchical materials. Its technical design has been tailored to offer complementary experimental modalities, enabling the investigation of materials across multiple length scales – from the nanometre scale using small- and wide-angle X-ray scattering (SWAXS) to the micro- and millimetre scale using full-field synchrotron X-ray microtomography (SRµCT). The beamline is equipped with a 3 m-long room-temperature in-vacuum undulator, with a period length of 17 mm and maximum effective deflection parameter of *K* = 1.89 at the minimum magnetic gap of 4.5 mm. The fifth to 13th harmonics of the undulator are used to operate within the energy range of 8–25 keV. The harmonic peaks exhibit a narrow energy profile (Δ*E* ≃ 100 eV), which facilitates quantitative analysis of the collected data.

ForMAX can operate with either a double-crystal monochromator (DCM) or a double-multilayer monochromator (MLM), depending on the needs of the experiment. Photon-demanding experiments, such as time-resolved experiments, benefit from the higher photon flux provided by the MLM, due to its larger bandpass (Δ*E*/*E* ≃ 1%). This configuration yields a beam size of 1.3 mm × 1.5 mm in the sample position, with a measured flux of 10^14^ to 5 × 10^14^ photons s^−1^ in the energy range of 9–20 keV at the minimum undulator gap. The photon flux is distributed over a narrow energy range, resulting in a high spectral flux density. As an example, at 16.5 keV with the smallest insertion-device gap, the photon flux distribution has a full width at half-maximum of 100 eV. This high spectral density makes ForMAX an ideal environment for deploying XMPI, as discussed in the following section, and enables the study of phenomena that remain inaccessible with conventional time-resolved tomography.

### 2.2. The XMPI setup

**Figure 1:**
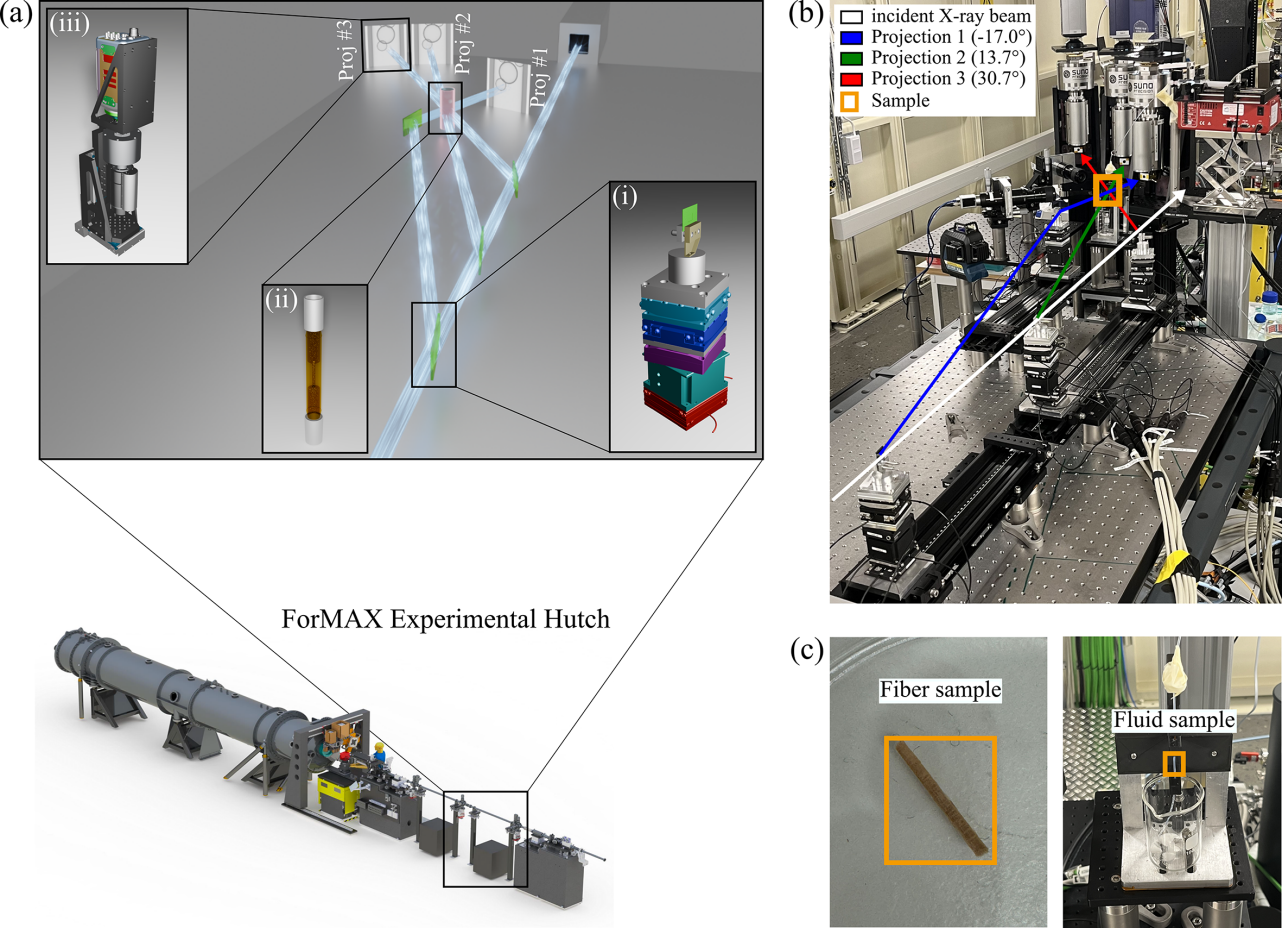
The XMPI setup at ForMAX. (*a*) The setup consists of beam splitters mounted on nanopositioners (i) for the generation of beamlets through spectral and amplitude splitting, that intersect at the sample environment position (ii), and an indirect detector system for each projection (iii). This illustration showcases an example study where the dynamics of interest concern the flow of particles through a capillary. The setup was commissioned upstream of the main sample station of ForMAX by modifying the box-marked segment in the beamline sketch. (*b*) A photograph of the setup in the ForMAX beamline with annotated projections. (*c*) Examples of samples that can be examined using XMPI: fiber (left) and fluid samples (right).

The XMPI setup consists of three major components, depicted in Fig. 1 ▸(*a*): (i) beam splitters for the generation of beamlets, *i.e.* sub-fractions of the direct incident beam that travel in air and intersect at the (ii) sample environment position, providing simultaneous, angularly resolved illumination, and (iii) an indirect detector system for each projection (Asimakopoulou *et al.*, 2024 ▸). The splitting setup is inspired by interferometers, split-and-delay lines, and specific setups for single-shot imaging as shown by Oberta & Mokso (2013 ▸), Mokso & Oberta (2015 ▸). The setup was commissioned at a dedicated section of the beamline, located upstream of the main endstation. The setup was installed by removing the vacuum pipes and installing two optical tables: one hosting the beam splitters and one for the sample environment and detectors, as illustrated in Fig. 1 ▸(*b*). Two examples of samples that can be examined using XMPI are shown in Fig. 1 ▸(*c*).

#### 2.2.1. Crystals

The XMPI setup aims to generate multiple illumination viewpoints on the sample position. This is achieved by employing perfect crystals for the generation of beamlets from an incident X-ray beam via two different splitting schemes: spectral and amplitude splitting. The former refers to the redirection of a portion of the incident beam’s spectrum, which has a bandwidth Δ*E* around its central energy, to a new direction due to diffraction by a crystal. This occurs when the crystal is oriented to fulfill the Bragg or Laue condition (Voegeli *et al.*, 2023 ▸; Villanueva-Perez *et al.*, 2023 ▸). The Bragg or Laue condition is fulfilled when the incident X-ray beam intersects a family of lattice planes at a specific angle of incidence θ_B_ with respect to the beam direction and is dependent on the crystal material, the lattice plane and the beam energy. The redirected portion of the beam (beamlet) travels at an exit angle of 2θ_B_. On the other hand, amplitude splitting refers to the positioning of the crystal so that this condition is only fulfilled for a portion of the beam’s footprint, *i.e.* only part of the crystal is in the beam path, allowing the rest of the beam to propagate unhindered (Roling *et al.*, 2012 ▸). This strategy avoids absorption losses, facilitating higher acquisition rates, albeit at the cost of decreasing the field of view (FoV). The feasibility of performing fast 3D imaging experiments with these splitting schemes is influenced by several factors, most importantly: stable and homogeneous illumination, which is essential for achieving sufficient image quality, photon flux, which determines signal-to-noise ratio and temporal resolution, and angular spacing of the viewpoints, which affects the quality of the volumetric reconstruction. These aspects will be elaborated in the following, beginning with an emphasis on the quality of the crystals used to generate the beamlets.

Stable and homogeneous beamlet illumination is inherently dependent on the quality of the beam splitters. Perfect, dislocation-free crystals have been shown to offer the best performance and were therefore used for the fabrication of the beam splitters employed in this work. Additionally, the fabrication protocols can influence the choice of materials, as well as the specifications for their size and thickness. While the detailed fabrication process is beyond the scope of this work, we emphasize that it plays a critical role in the performance of the XMPI setup. Even when the fabrication of the crystal is deemed successful, *i.e.* the crystals are mostly dislocation-free, radiation damage on the beam splitters during their exposure to X-rays can significantly degrade the image quality. To maintain stable and homogeneous illumination for high-quality imaging, thermal effects have to be considered. These effects can manifest as microscopic and macroscopic morphological changes in the crystal. Therefore, materials with high thermal conductivity and high melting point are preferred, as they are more resistant to heat-induced deformation and radiation damage, ensuring long-term stability and performance of the XMPI setup.

The photon flux carried by the beamlets is influenced mainly by the beam and crystal properties. Regarding the former, to increase the flux carried by the beamlets, it is desirable to have a beam with small divergence, due to the finite angular acceptance (Darwin width) of the crystal, and high spectral flux density, as typically illustrated in DuMond diagrams (Authier, 2001 ▸). Regarding the crystal properties, one has to optimize the choice of crystal characteristics such as the material’s atomic number (*Z*), diffraction order and the layout-introduced effects, *i.e.* flux losses from transmission through elements placed upstream of each beam splitter. The atomic number of the crystal material affects the diffraction intensity (and thus the beamlet flux) since materials with higher *Z* contain more electrons, increasing the probability of X-ray diffraction within the crystal’s extinction length (Authier, 2001 ▸). This flux directly affects the utilized analog-to-digital converter (ADC) range of the detector: higher diffraction efficiency, *i.e.* a larger amount of diffracted photons with respect to the incident direct beam, allows for higher acquisition rates while maintaining full detector performance. However, while high-*Z* materials offer stronger diffraction intensities, they also exhibit short extinction lengths and greater beam attenuation. This is particularly important because some of the beam splitters are arranged sequentially along the beam path, as will be discussed later. These competing properties necessitate the use of thin implementations of high-*Z* materials to balance diffraction efficiency and transmission. In addition to material properties, diffraction efficiency is influenced by the Darwin width and the diffraction order of the crystal. The former is an individual property of crystal materials, and a larger Darwin width will lead to diffraction of a larger bandwidth of the spectrum. Higher diffraction orders enable exploration of larger angles, which is desirable for maximizing the angular spacing between beamlets. However, this comes at the cost of reduced diffraction efficiency, especially due to the horizontal polarization of synchrotron and X-ray free-electron laser sources, which leads to significantly lower efficiency at Bragg angles near 45° (Authier, 2001 ▸).

To address these constraints, we currently employ four beam splitters in our layout, as illustrated in Fig. 1 ▸: three beam splitters are placed sequentially along the primary beam path, and a fourth beam splitter is placed off-axis with respect to the primary beam to redirect the beamlet generated by the first beam splitter towards the sample position, thereby increasing the angular spacing between the beamlets. All beam splitters are oriented to fulfill the Bragg condition, except for the second sequential beam splitter, which is oriented to fulfill the Laue condition.

The previously discussed aspects were taken into account when choosing which crystals and diffraction planes to utilize in our XMPI setup. As materials, we selected Ge and Si crystals, as these are among the most accessible materials that possess high purity and exhibit minimal dislocations. To generate the projections, all beam splitters employed spectral splitting, the crucial concept of XMPI. Additionally, the first crystal was also an amplitude splitter to reduce absorption losses. We considered the Darwin widths of different diffraction planes to obtain sufficient flux on each projection, while at the same time being able to position the beam splitters accurately with the precision accessible with the nanopositioners on which they are mounted. Details of the selected crystals for the different projections together with their corresponding Darwin widths are shown in Table 1 ▸.

The precise positioning of the beam splitters is crucial to successfully perform XMPI experiments. Each crystal must simultaneously satisfy its Bragg condition and be accurately aligned so that all beamlets intersect at a common point in space. Therefore, we employ a stack of six nanopositioners for each beam splitter, providing us with six degrees of freedom (three translations, one rotation, two goniometers). The translational stages provide a 1 nm closed-loop positioning resolution with the horizontal (vertical) stage covering a total travel range of 30 mm (8 mm). The rotation stage is capable of continuous rotation with 0.01 m° (0.036 arcsec) resolution. The goniometers are able to move ± 5° with 1 µ° (0.0036 arcsec) resolution. All positioners are operated in closed-loop mode via manufacturer-provided application programming interfaces (APIs), which are integrated into a custom Python control script.

#### 2.2.2. Detectors

The detection of each beamlet after the sample is performed using indirect detectors – detectors that first convert the X-rays to visible light, magnify the visible light through an optical microscope and then record the images with a visible-light sensor. The detector setup is modular, allowing for different configurations of scintillators, magnification optics and cameras, to accommodate a wide range of experimental requirements in terms of acquisition rate, spatial resolution and FoV of interest. The detector positions can be adjusted for each experiment as needed. They are positioned at the angle of the corresponding projection, *i.e.* 2θ_B_ with respect to the beam axis, as listed in Table 1 ▸. The distance between the sample and the detector may vary between projections, as it is primarily determined by the available physical space, typically ranging from 350 mm to 450 mm.

The scintillator is mounted in a dedicated unit, designed to accommodate scintillators of 8 mm × 8 mm in size and up to 300 µm thick. In this work, we use a 250 µm-thick GaGG+ scintillator, known for its excellent high yield (45000 photons MeV^−1^), chosen to match the depth of focus for our desired spatial resolution of ∼8 µm. The microscopes are equipped with an objective holder featuring a motorized focusing unit, compatible with 5× to 20× objectives covering a broad wavelength spectrum, providing the flexibility to employ various cameras with different sensor types and scintillators. A high-resolution 5× objective is typically used. The focus unit is driven by a two-phase stepper motor, offering a 10 mm travel range with 1 µm resolution. Each microscope includes a tube lens holder with a motorized camera rotation unit. The nominal design is for a 1× tube lens. The rotation unit covers a range of 90 mrad with an accuracy of 0.02 mrad, and supports C- and F-mount cameras.

Additionally, the microscopes are mounted on a motorized positioning system for linear motion perpendicular to the beam-incidence axis and along the vertical axis, with travel ranges of 40 mm and 26 mm, respectively, and a resolution of 5 µm.

The choice of camera is determined by the spatial and temporal resolution needs for the given experiment. Commercial cameras often necessitate a trade-off between spatial and temporal resolution (Olbinado *et al.*, 2017 ▸). According to the Nyquist–Shannon theorem (Thapa *et al.*, 2015 ▸), a feature can be resolved when the sampling rate is at least twice the highest spatial frequency – corresponding to two pixels per feature. Thus, higher spatial resolution requires smaller pixel sizes, which in turn have a lower signal-to-noise ratio and demand higher flux or longer exposure times, effectively limiting the achievable temporal resolutions. Conversely, high temporal resolution requires high pixel sensitivity and fast readout schemes, with the latter often achieved by decreasing the effective sensor area at higher acquisition rates.

In this work, we deployed the XMPI microscopes with two camera systems, the Photron Nova S16 for high temporal resolution and the Andor Zyla 5.5 for high spatial resolution. Their specifications are summarized in Table 2 ▸. The Photron Nova S16 supports full-frame acquisitions (1024 × 1024 pixels) at up to 16 kHz, and can reach 1.1 MHz at a reduced frame (128 × 16 pixels). The Andor Zyla 5.5 can reach 49 Hz with its full sensor (2560 × 2160 pixels).

We have performed a detailed analysis of the spatial resolution of the optical system using the Photron Nova S16 at 40 kHz, with the 250 µm-thick GaGG+ scintillator and 5× objective at an energy of 16.3 keV. We concluded that the Nyquist–Shannon theorem is the limiting factor, providing a spatial resolution of 8 µm (2 × 4 µm) (Yao *et al.*, 2024 ▸). The same considerations would mean a spatial resolution of 2.6 µm (2 × 1.3 µm) for the Andor Zyla 5.5.

XMPI provides simultaneous illumination of the sample and, therefore, a synchronous camera acquisition scheme is required. A hardware trigger signal initiates the acquisition of each camera. This trigger can be configured to be issued (i) from the sample if events of interest can be used as a trigger, *e.g.* tensile load value exceeding a minimum, or (ii) from an external input like a hardware button trigger. The Zyla cameras are fully integrated in the ForMAX signal control system, ensuring synchronized hardware triggering using a PandABox (Zhang *et al.*, 2017 ▸). The Photron cameras were operated in a chain configuration: one camera received the trigger signal and acted as the primary transmitter, triggering the acquisition of the two other cameras, which acted as receivers.

In this work, we refer to the Photron Nova S16 configuration as the ‘high temporal resolution’ setup, and the Andor Zyla 5.5 configuration as the ‘high spatial resolution’ setup.

#### 2.2.3. Sample

XMPI is compatible with a plethora of scientific samples and sample environments. The X-ray energy used in an experiment is determined by the contrast and flux requirements of the sample under investigation, while the physical footprint of the sample environment dictates the necessary spacing between the beam splitters. To ensure accurate alignment, the region of interest (ROI) within the sample is positioned at the beamlet intersection point using a stack of three linear motors – one for vertical and two for horizontal adjustments.

### 2.3. Data processing and reconstruction

To reconstruct the 3D volume from the three recorded projections, we start by pre-processing the data. We perform conventional flat-field correction on the projections to reduce the inhomogeneities caused by the beam itself. For this, we record a series of flats, *i.e.* images without the sample in the beam, and average them. Then, we divide the projections by the average flat field (Van Nieuwenhove *et al.*, 2015 ▸).

For the actual reconstruction, we face the challenge of having a sparse number of views available. In conventional tomography, the Crowther criterion is used to determine the number of required projections *N*_θ_ based on the number of horizontal pixels *N*_*x*_ (Jacobsen, 2019 ▸): 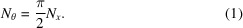
In the case of 1024 pixels, this corresponds to ∼1600 projections instead of the three projections offered with the presented layout of XMPI. Consequently, traditional reconstruction algorithms such as filtered back projection (FBP) are unable to accurately reconstruct the volume. To perform 4D reconstructions (3D + time) from a sparse number of projections, one can use the recently developed self-supervised deep-learning tools 4D-ONIX and X-Hexplane that include the physics of X-ray interaction with matter.

Both algorithms directly reconstruct the 4D volume. 4D-ONIX operates by (i) integrating X-ray propagation physics into the model, (ii) employing a continuous representation of the sample, which describes the refractive index as a function of spatial and temporal coordinates, (iii) learning the latent features of the sample by generalizing across different experiments of similar sample dynamics, and (iv) using adversarial learning to enforce consistency between real and predicted projections (Zhang *et al.*, 2023 ▸; Zhang *et al.*, 2025 ▸; Yao *et al.*, 2025 ▸). X-Hexplane operates in a similar manner but represents 4D as a factorized combination of orthogonal spatial and spatiotemporal feature planes, *i.e.* three spatial (XY, YZ, ZX) and three spatiotemporal (XT, YT, ZT) (Cao & Johnson, 2023 ▸). By employing this tensorial representation, X-Hexplane improves memory efficiency and computational performance compared with 4D-ONIX. The detailed working principles of both algorithms are beyond the scope of this work, and the interested reader is referred to the works of Zhang *et al.* (2025 ▸) and Hu *et al.* (2025 ▸).

**Figure 2:**
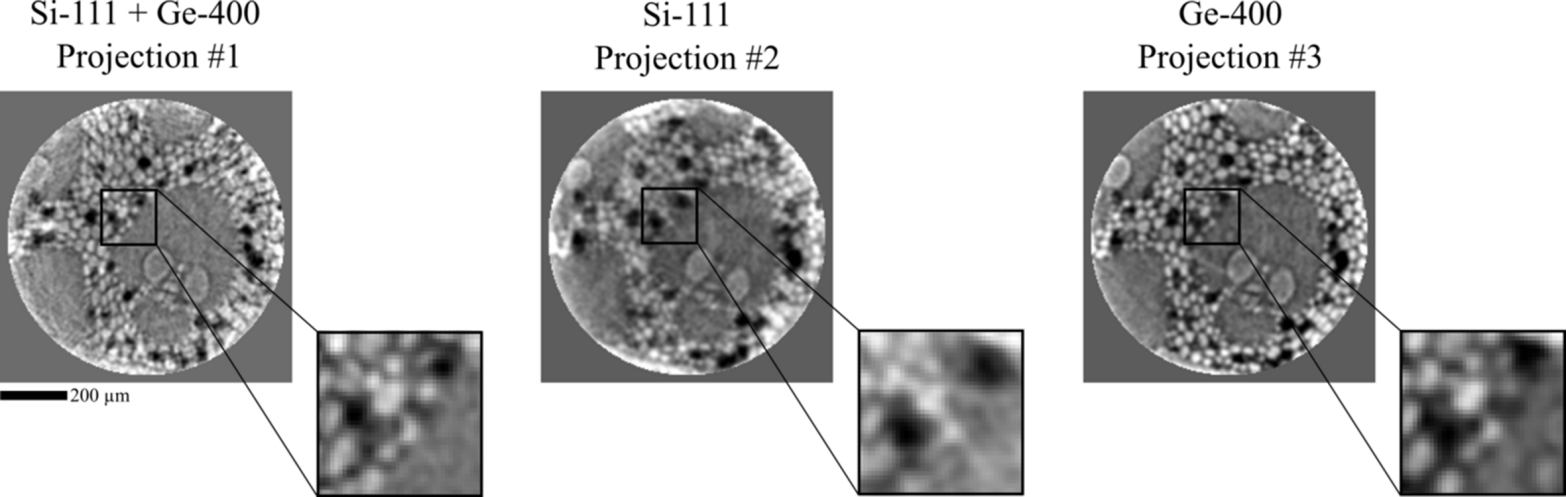
Reconstructed tomography slice of a bamboo rod recorded with each beamlet individually. The dataset was acquired with an acquisition speed of 6 kHz and a rotation speed of 72° per second. For the reconstruction, 349 projections over 180° were used following the Crowther criterion.

As of now, 4D-ONIX and X-Hexplane have been successfully applied to reconstruct simple dynamics, such as droplet collisions, in 4D. For a reconstruction of more complex sample systems, additional physics knowledge, such as support constraints or equations describing the underlying physical processes, can be highly beneficial. Having more prior information available, *e.g.* from several experiments, and/or a larger number of projections, also improves the reconstruction (Zhang *et al.*, 2025 ▸; Yao *et al.*, 2025 ▸). Alternatively, we are working on enriching the spatial information by combining a sufficiently slow rotation with XMPI, if the sample process in question allows for it, thereby helping with the reconstruction (Hu *et al.*, 2025 ▸). We can also perform standard tomography to constrain either initial or final sample states. In Fig. 2 ▸ we demonstrate the quality of the beamlets by performing tomography of a bamboo rod with each of the three beamlets (Si-111 + Ge-400, Si-111 and Ge-400) with an acquisition rate of 6 kHz and rotation speed of 72° per second, when using 349 projections over 180° for the reconstruction, following the Crowther criterion. It should be noted that the tomogram acquired with the Si-111 appears more blurry due to Laue diffraction accompanied by the Borrmann triangle effect, as will be discussed in Section 4.

Depending on the goal of the experiment, a 4D reconstruction might not be necessary. Another example of employing XMPI is particle tracking, where two projections are sufficient in order to identify individual particles and study their behavior (Rosén *et al.*, 2024 ▸).

## 3. Examples of scientific applications enabled by XMPI

Scientific applications with (i) sensitive dynamics, such as fluidic samples and wood fibers, and (ii) complex sample environments are two cases of the previously mentioned studies that cannot be fully explored with *in situ* and operando time-resolved tomography. The potential of XMPI lies in probing unexplored phenomena in such dynamics, and in this work we employ XMPI for the study of (i) loading properties of wood fibers under different stages of kraft cooking and (ii) particle suspension in multiphase flows.

### 3.1. Samples

#### 3.1.1. Wood fibers’ loading properties

With increasing interest in replacing plastic-based materials, wood fibers are gaining importance in construction, pulp and paper, and advanced technology. To advance wood-based materials towards such applications, it is crucial to understand their mechanical behavior under external stress. XMPI offers an opportunity to study in 3D the deformation process and breakage under load at spatiotemporal resolutions not possible with state-of-the-art methods. Moreover, it circumvents the misinterpretation of fiber mechanical behavior by using one projection.

Here, we focused on loading properties of spruce wood fibers under different stages of kraft cooking (Dang *et al.*, 2016 ▸), a process typically used in industries to extract cellulose fibers. The sample size was roughly 500 µm in both width and thickness, while the length was adjusted to 1 cm. Considering that the average size of a spruce fiber is between 30 and 50 µm in diameter, the dynamics of more than 10 by 10 fibers can be observed simultaneously, where the deformation of wood fiber walls (around 5 µm thick) and crack propagation within them would be detected. We needed to prioritize high temporal resolution during the measurements, since the events of interest were expected to occur on timescales shorter than 1 ms. To this end, the high temporal resolution configuration of XMPI was deployed for 12.5 kHz acquisitions, at an energy of 16.5 keV.

#### 3.1.2. Multiphase flows

Understanding the behavior of multiphase flows is essential due to their prevalence in both natural and industrial processes, motivating the need to acquire volumetric 4D information (3D + time) without disruptive forces. For this reason, we performed XMPI experiments to study multiphase flow properties via particle tracking, only requiring two projections. For this purpose, we chose Projections #1 and #3 to maximize the angular coverage. We examined the motion of silver-coated hollow glass spheres (SHGS) with a diameter of 10 µm through a glycerol solution, thereby minimizing gravity-induced effects on the flow. To accurately monitor the particles’ flow on the micrometre scale, we prioritized achieving a high spatial resolution by deploying the high spatial resolution XMPI configuration while compromising at lower temporal resolutions with acquisitions of 40 Hz, exploring flow rates of 0.1, 0.2 and 0.5 ml h^−1^. To optimize the contrast within the flow system, we chose an energy of 16.5 keV (Rosén *et al.*, 2024 ▸).

Such volumetric and time-resolved X-ray studies represent a critical advancement, paving the way for future experiments that can explore previously inaccessible research questions regarding, for example, blood flow properties and food and material processing.

## 4. Discussion

We deployed XMPI at ForMAX for two experimental studies. The studies were performed at 16.5 keV with two configuration setups, allowing us to explore distinct spatiotemporal regimes. The experimental setup, along with indicative data, is presented in Section 4.1, followed by the discussion of current challenges and limitations in Section 4.2.

### 4.1. Current possibilities of XMPI at ForMAX

Our experiments demonstrated the potential of XMPI experiments at ForMAX on studies with high temporal (Experiment #1) and high spatial (Experiment #2) resolution requirements, utilizing the detector’s full or near-full ADC range. The utilized ADC range serves as an indicator of photon statistics of our detector, helping us to optimize illumination by adjusting the acquisition rate and/or applying filtering.

#### 4.1.1. Experiment #1

The high temporal resolution configuration of XMPI at ForMAX was deployed with the Photron Nova S16 cameras (12 bit) for the imaging of wood fiber breakage, which occurs on sub-millisecond timescales and requires above kHz acquisitions for capturing the event of interest. The configuration offers a spatial resolution of approximately 8 µm on each projection (Section 2.2.2). We deployed XMPI with three projections with the use of four beam splitters (Table 1 ▸).

**Figure 3:**
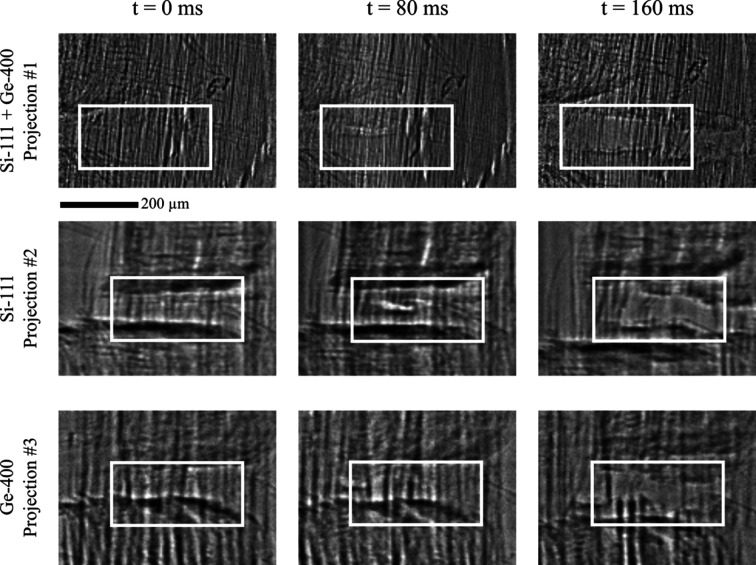
XMPI projections of wood fiber failure recorded at 12.5 kHz with an energy of 16.5 keV. The failure region is highlighted by a box. Three representative time stamps were selected (0 ms, 80 ms and 160 ms). The projections were flat-field-corrected using conventional flat-field correction. Projection #1 with −17.0°, #2 with 13.7° and #3 with 30.7° with respect to the primary beam axis were generated with a recombiner (Si-111 + Ge-400), Si-111 and Ge-400, respectively.

Breakage of wood fibers is a dynamic process that requires this level of spatiotemporal resolution, as discussed in Section 3.1.1, and was thus deemed ideal for the demonstration of the high temporal capabilities of XMPI. The evolution of the process was recorded successfully from three angularly spaced viewpoints over several seconds, with a temporal resolution of 160 µs assuming Nyquist resolution. Fig. 3 ▸ presents a selection of frames acquired during the experiment. The utilized ADC of the recorded data ranged from 10 to 12 bits.

#### 4.1.2. Experiment #2

**Figure 4:**
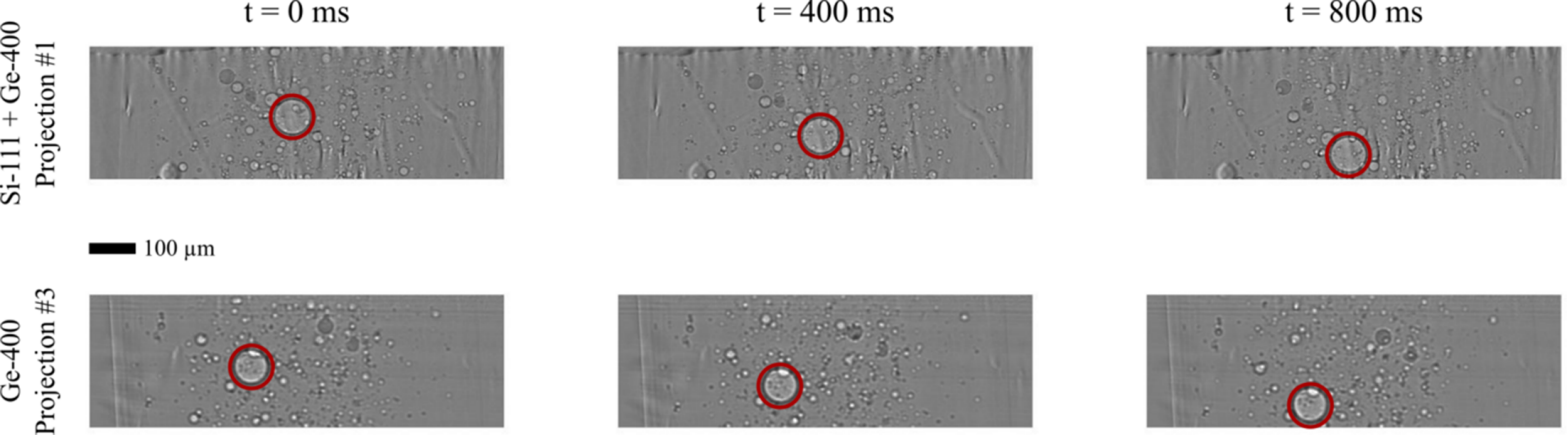
XMPI projections of SHGS suspended in glycerol recorded at 40 Hz with an energy of 16.5 keV. Three representative time stamps were selected (0 ms, 400 ms and 800 ms). The projections were flat-field-corrected using conventional flat-field correction. Projection #1 with −17.0° and #3 with 30.7° with respect to the primary beam axis were generated with a recombiner (Si-111 + Ge-400) and Ge-400, respectively. The same particle is highlighted in red in each frame to illustrate its movement with time.

The high spatial resolution configuration of XMPI at ForMAX was deployed with the Andor Zyla 5.5 cameras (16 bit) for the imaging of multiphase flows. The configuration enables the resolution of features down to 2.6 µm, meeting the requirements for this sample study. Images were recorded at an acquisition rate of 40 Hz with Projections #1 and #3, as listed in Table 1 ▸. Fig. 4 ▸ shows selected frames from the two projections, with SHGS particles suspended in glycerol as the sample. The full ADC range of the detector system was utilized in this configuration. The detailed analysis of the flow properties is out of the scope of this work but is discussed by Rosén *et al.* (2024 ▸).

### 4.2. Splitting limitations

We are continuously striving to improve the XMPI setup, further developing and optimizing the instrumentation. Improving the hardware will lead to higher-quality data and will enable better analysis methods, most importantly volume reconstructions, where needed.

**Figure 5:**
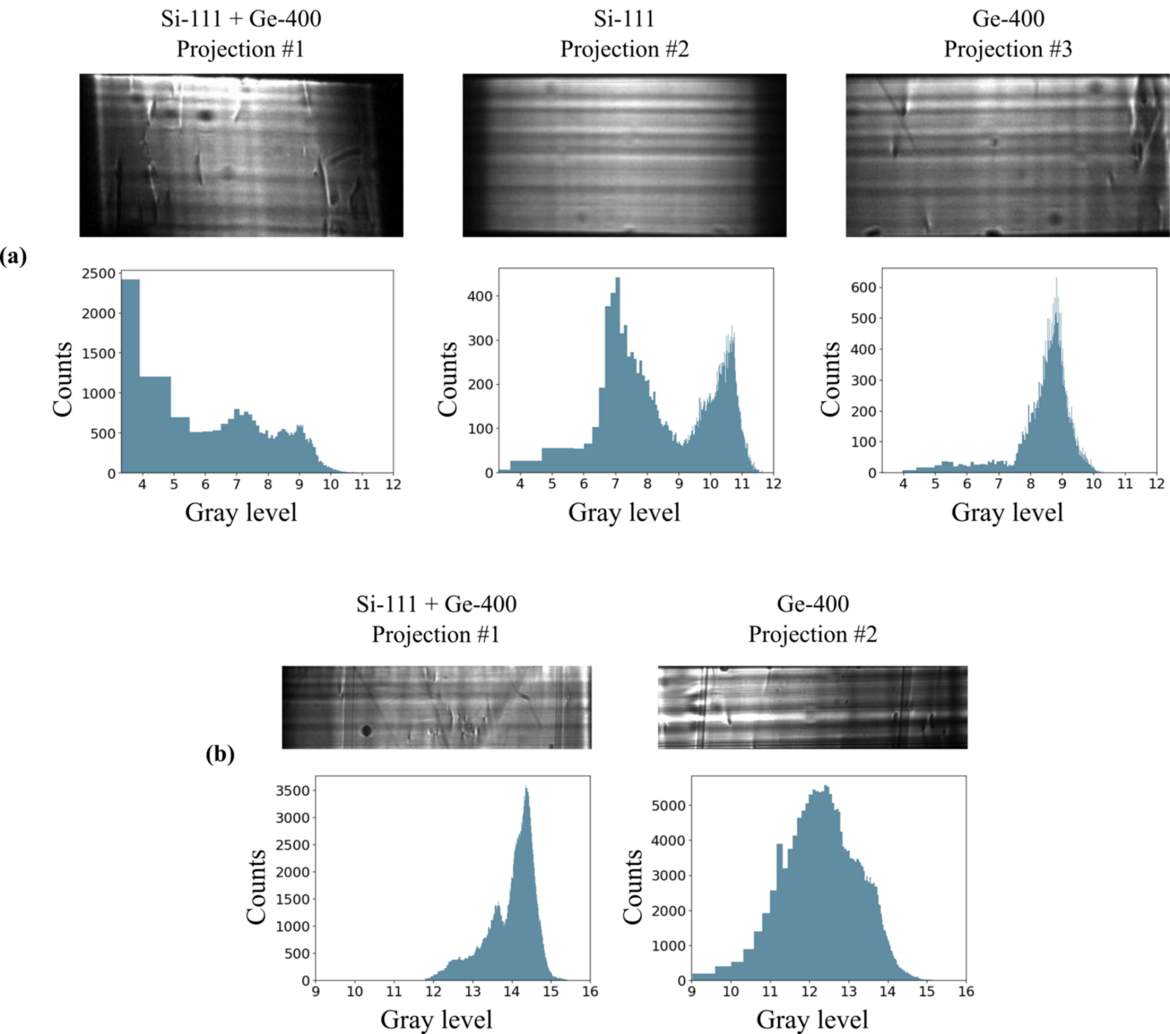
Flat-field images of two different experiments acquired at 12.5 kHz (*a*) and 40 Hz (*b*) with corresponding histograms. The *x* axis is log-scaled with base 2, and the tick labels indicate the exponent. (*a*) The high temporal resolution configuration covers an ADC range of 12 bit (Projection #1), 12 bit (Projection #2) and 11 bit (Projection #3). The full ADC range of the Photron Nova S16 is 12 bit. (*b*) The high spatiotemporal resolution configuration covers an ADC range of 16 bits for both projections, *i.e.* the full ADC range of the Andor Zyla 5.5.

High-quality projections, homogeneous illumination with as few artifacts as possible, are required by XMPI. These strongly depend on the beam splitters. Such a homogeneous intensity can be disturbed because of diffraction at slightly different angles in the active area of the crystal, induced by imperfect crystals or non-optimal clamping of the crystals. In fact, the fabrication of perfect crystals that satisfy our requirements when it comes to dimensions and purity, as well as the absence of dislocations, is challenging. Difficulties with inhomogeneous illumination are depicted in Figs. 5 ▸(*a*) and 5 ▸(*b*), where flat-field images of the projections with their corresponding histograms are presented. Some parts of the flats are saturated due to uneven illumination, resulting in certain areas of the image appearing significantly brighter than others. An approach to overcome fabrication challenges is to employ Laue rather than Bragg diffraction. Using a beam splitter in the Bragg configuration requires a larger active area of the crystal, as the angle between the beam splitter and the direct incident beam is small, *i.e.* the beam has a large footprint, and more surface is needed to diffract the whole beam. In contrast, beam splitters used in symmetric Laue geometry can be exposed to a smaller beam footprint, relaxing the requirements for the size of the active area. This is important because it is less demanding to manufacture smaller crystals with the same technical specifications, which reduces the risks of imperfections. However, it should be noted that Laue diffraction introduces some blurring in the image due to the Borrmann triangle effect (Authier, 2001 ▸; Asimakopoulou *et al.*, 2024 ▸). Moreover, to avoid strains on the crystal surface, it is important to optimize clamping schemes and consider strain-relief cuts (Bellucci *et al.*, 2024 ▸).

Here, we used the beam splitter of Projection #2 in symmetric Laue geometry. We employed a silicon crystal formed as a thin membrane supported by an integral frame. The membrane was fabricated by adapting processes developed for crystals used to study high-energy particle–crystal interactions (Mazzolari *et al.*, 2013 ▸; Scandale *et al.*, 2014 ▸; Germogli *et al.*, 2015 ▸). A silicon-oxide hard mask was deposited on 100 mm-diameter, 0.5 mm-thick silicon wafers through low-pressure chemical vapor deposition techniques and patterned photolithographically. The exposed silicon was then thinned in an isotropic wet etchant (Madou, 2011 ▸). In our process, the attainable active area was limited to a few tens of mm^2^, and the thickness uniformity across the aperture was ∼5 µm. Isotropic undercut and etch-bath hydrodynamics imposed practical limits on window size and thickness uniformity (Kuiken, 1984 ▸). After thinning, the hard mask was removed by wet etching, and the wafer was diced to release individual crystals. The frame enabled safe handling and, crucially, prevented clamp-induced stresses from propagating into the active diffracting area. To decouple the clamp from the active region, we machined strain-relief cuts in the frame (Bellucci *et al.*, 2024 ▸), forming a slotted compliant suspension that localized mechanical loads due to clamping and mitigated stress transfer to the membrane.

The high photon flux required to perform XMPI experiments with sufficiently intense diffracted X-ray beams will induce heat load on the beam splitters. Depending on the material and photon flux, this can appear as warping issues or vibrations, visible at short timescales, or, in extreme cases, as morphological changes of the crystal. Therefore, one might consider thermalization and/or cooling of the crystals when acquiring images.

Another limitation of XMPI is the angular coverage of the setup. Obtaining higher diffraction angles requires higher diffraction orders, which comes with the cost of lower diffraction efficiency (Authier, 2001 ▸; Liang *et al.*, 2023 ▸; Bellucci *et al.*, 2024 ▸). In addition, XMPI requires a volume reconstruction from a sparse number of projections, something that is possible with our algorithms for simple dynamics. However, more projections would help to improve the reconstructions further. In principle, the number of projections can still be increased (Voegeli *et al.*, 2023 ▸; Voegeli *et al.*, 2024 ▸; Yashiro *et al.*, 2024 ▸) but, at a certain point, we will reach a limit in terms of physical space available to fit our equipment, remaining bandwidth of the incident’s beam undulator spectrum for spectral splitting (smaller Darwin widths will enable more projections at the cost of achievable spatiotemporal resolution per projection) and remaining beam size when employing amplitude splitting. Therefore, we are also integrating the possibility of an additional slow rotation of the sample (if the process allows it) in our XMPI experiments, thereby increasing the number of acquired projections. Finally, the performance of our algorithms in the volumetric time-resolved reconstruction of dynamic processes can be further increased by the 3D characterization (tomography) of the initial and final states of the studied samples, whenever possible.

## 5. Conclusion and outlook

In this work, we presented the commissioning and potential of our XMPI setup at the ForMAX beamline at MAX IV. XMPI is a technique that can be used to retrieve 4D information on processes of interest, without the need for sample rotation, a feature that overcomes known limitations in present state-of-the-art methods such as tomography. This novelty enables temporally resolved volumetric imaging of presently unexplored phenomena in rotation-sensitive samples, samples that require complicated study environments, samples with fast (above kHz) dynamics, or single-shot phenomena. In XMPI, several projections are acquired simultaneously by splitting the direct incident X-ray beam into beamlets that intersect at the sample position, by employing beam splitters. The beam quality at ForMAX provided a unique environment for the successful commissioning of the technique due to its high spectral flux density.

We deployed XMPI in two different spatiotemporal regimes: a high temporal resolution configuration for at least 12.5 kHz acquisitions with the ability to distinguish 8 µm features and a high spatial resolution configuration for distinguishing 2.6 µm features with 40 Hz acquisitions, in the recorded projections. The commissioning of the two configurations was done during our reported work on Experiment #1, for the study of wood fiber failure when exposed to external forces, and Experiment #2, for the study of opaque multiphase particle flows, where we performed in-flow particle tracking.

We discussed the presently identified main challenges of the technique, *i.e.* (i) the fabrication of perfect crystals, (ii) strains and artifacts on the crystals, and (iii) a limited number of projections. We have identified the difficulty in addressing (i), as we depend on having dislocation-free, perfect crystals that are large enough to capture the full X-ray footprint and have a thickness that takes account of the extinction length and still lets photons transmit towards the next beam splitter. Regarding (ii), we conclude that alternative clamping methods and strain-relief cuts, as well as employing beam splitters in Laue geometry, will perform better. Furthermore, thermalization and cooling mechanisms should be considered to decrease the heat load on the crystals. To tackle (iii), we have the possibility to implement a slow rotation of the sample to acquire more projections, which will help with the volume reconstruction. In the future, we plan to test more materials as beam splitters, allowing us to explore different geometries and diffraction efficiencies. Moreover, we may consider more efficient signal detection schemes with a higher signal-to-noise ratio using direct conversion detectors, instead of scintillator-based microscopes, relaxing requirements when it comes to the flux of the beamlets without compromising on spatiotemporal resolution.

In conclusion, XMPI presents exciting opportunities for 4D imaging, enabling the study of phenomena that remain unexplored due to the limitations of present state-of-the-art methods. The deployment of XMPI at ForMAX allowed the exploration of its full potential at the beamline and we foresee that it has potential for applications that span a broad spectrum of research areas, including blood flow analysis, additive manufacturing, fracture behavior in composites, failure mechanisms in fibers and the compression dynamics of foams, to name just a few. This advancement opens up a new era for 4D imaging, paving the way for discoveries across diverse scientific research questions.

## Figures and Tables

**Table 1:** Experimental parameters of the two XMPI experiments performed at ForMAX, MAX IV

Projection	Parameters	Values
	Energy	16.5 keV
	Angular coverage	48°

#1	Beam splitter	Si + Ge
	Diffraction mode	Bragg
	Out-of-plane	111 + 100
	Diffraction plane	111 + 400
	Darwin width	3.2 arcsec and 3.0 arcsec
	Angle with respect to beam axis	−17.0°

#2	Beam splitter	Si
	Diffraction mode	Laue
	Out-of-plane	111
	Diffraction plane	111
	Darwin width	3.2 arcsec
	Angle with respect to beam axis	13.7°

#3	Beam splitter	Ge
	Diffraction mode	Bragg
	Out-of-plane	100
	Diffraction plane	400
	Darwin width	3.0 arcsec
	Angle with respect to beam axis	30.7°

**Table 2:** Specifications of the cameras deployed for the XMPI experiments discussed in the present paper

Camera	Sensor size (W × H)	Pixel size (W × H)	ADC
Photron Nova S16	1024 pixels × 1024 pixels	20 µm × 20 µm	12 bit
Andor Zyla 5.5	2560 pixels × 2160 pixels	6.5 µm × 6.5 µm	16 bit

## Data Availability

The data supporting the results of this study are available within this article and through the Zenodo repository https://zenodo.org/records/18174214?token=eyJhbGciOiJIUzUxMiJ9.eyJpZCI6IjU2YWIxMTFlLWU4NDUtNDM1YS05MjM4LWY3NDRiYmFmMDBkMiIsImRhdGEiOnt9LCJyYW5kb20iOiI5YzQ4M2Q3MzY1M2ZmMDFiN2RiNWYzYTEwNDU0NWM0YSJ9.DO6JcyfKPU8NVoDn0G9tDfCE2EqD4agbPmQa89V6KmuqTxVYwl3tssPPhqIsi6DKtXb_HRTR4pWsudZW2T5_Wg.
